# Visual Diagnosis of the Varroa Destructor Parasitic Mite in Honeybees Using Object Detector Techniques

**DOI:** 10.3390/s21082764

**Published:** 2021-04-14

**Authors:** Simon Bilik, Lukas Kratochvila, Adam Ligocki, Ondrej Bostik, Tomas Zemcik, Matous Hybl, Karel Horak, Ludek Zalud

**Affiliations:** Department of Control and Instrumentation, Brno University of Technology, 61 600 Brno, Czech Republic; bilik@feec.vutbr.cz (S.B.) kratochvila@feec.vutbr.cz (L.K.); adam.ligocki@vutbr.cz (A.L.); bostik@feec.vutbr.cz (O.B.); zemcikt@feec.vutbr.cz (T.Z.); matous.hybl@ceitec.vutbr.cz (M.H.); zalud@feec.vutbr.cz (L.Z.)

**Keywords:** *Varroa destructor*, *Apis mellifera*, western honey bee, bee health monitoring, object detection, YOLO, SSD, deep learning

## Abstract

The *Varroa destructor* mite is one of the most dangerous Honey Bee (*Apis mellifera*) parasites worldwide and the bee colonies have to be regularly monitored in order to control its spread. In this paper we present an object detector based method for health state monitoring of bee colonies. This method has the potential for online measurement and processing. In our experiment, we compare the YOLO and SSD object detectors along with the Deep SVDD anomaly detector. Based on the custom dataset with 600 ground-truth images of healthy and infected bees in various scenes, the detectors reached the highest F1 score up to 0.874 in the infected bee detection and up to 0.714 in the detection of the *Varroa destructor* mite itself. The results demonstrate the potential of this approach, which will be later used in the real-time computer vision based honey bee inspection system. To the best of our knowledge, this study is the first one using object detectors for the *Varroa destructor* mite detection on a honey bee. We expect that performance of those object detectors will enable us to inspect the health status of the honey bee colonies in real time.

## 1. Introduction

**Figure 1:**
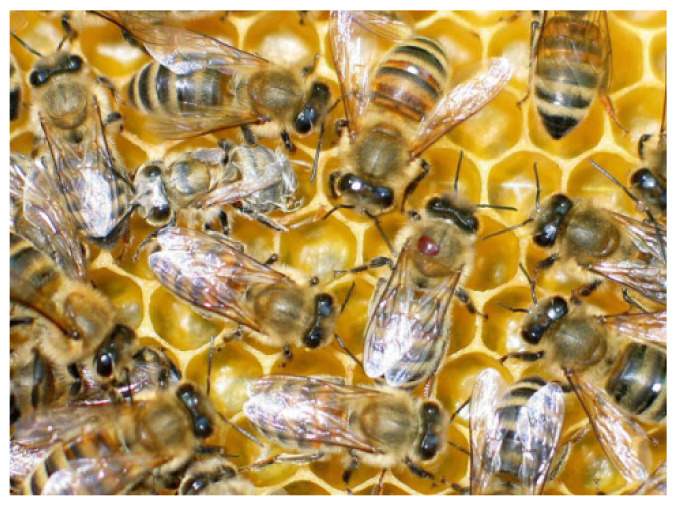
V.-mite on an adult bee worker (in the centre) [1] Reprinted from Publication Journal of Invertebrate Pathology, 103, P. Rosenkranz; P. Aumeier; B. Ziegelmann, Biology and control of Varroa destructor, 96–101, Copyright (2010), with permission from Elsevier.

*Varroa destructor mite* (V.-mite) is a honey bee (*Apis mellifera*) ectoparasite, which is spread around the whole world, except Australia. This parasite can be seen at Figure 1. It originated from East Asia and started to spread around the world around the half of the 20th century. Because of its East Asian descend and relatively recent arrival, the western honey bee is not adapted for this parasite. Due to this factor, periodic V.-mite monitoring and treatment has to be performed. The conventional monitoring methods include the optical inspection of the bee colony detritus (when the bee keeper searches detritus for the fallen mites), or the V.-mite isolation from the bee sample (for example powdering sample of the bees with sugar, which causes the mites to fall), but those methods are time consuming and labour intensive. The other disadvantage of these methods is, that only a part of the mites could be detected. The V.-mite also becomes resistant to the current medications, so new treatment methods and detection approaches for early infection warning have to be developed [1].

The authors of the paper [2] also identify the most common areas, where the V.-mite attach to the bee. It attacks the bees in the larval state and feeds on its fat tissue, mostly in the belly area. This causes development failures of the bee brood and its losses. Surviving bees are often deformed due to these failures and they could be harmed by secondary viral infections propagated by this mite as well (V.-mite is confirmed, or suspect to transmit at least 18 viruses) [2]. All these factors can cause serious, even fatal bee colony losses. Although the most critical are losses in the winter period, proper treatment has to be performed already in the late summer, so the bees spending winter are already not significantly affected by this mite.

Bee colony winter losses are also concerned in the work [3], which presents the results obtained from the monitoring of 1200 bee colonies. The authors prove connection of the V.-mite infestation to bee colony winter mortality, where the infestation level was statistically lower in the surviving colonies. The study shows, that the V.-mite infestation level of 10% (V.-mites over 100 bees) increases the risk of the bee colony collapse up to 20% and the infestation level of 20% increases this risk up to 50%. The results also show the correlation between the V.-mite infestation level, presence of several bee viruses in such infected colonies and also with the still unclear Colony Collapse Disorder, which caused huge losses in several states worldwide.

Liebig [4] considers a safe V.-mite infestation rate in an untreated bee colony up to 7%. The author shows the importance of a proper and well timed treatment, when even heavily infected colonies with infestation level up to 70% treated in late August could be saved with a single treatment, but colonies with infestation level over 25% that were treated in November are still in a significant danger of collapse.

In this paper we present a non-invasive computer vision (CV) based approach for the V.-mite monitoring, which can be later used as a foundation of a portable and low-cost online monitoring system. We believe, that this method could have the potential to detect more mites, compared to traditional bee keeping methods mentioned above. This could result in an early and more accurate V.-mite infestation detection and to the possibility of a faster and more proper treatment.

Our paper is structured as follows: In the first part, we bring a short overview of the existing bee monitoring methods with the emphasis on the existing V.-mite automatic detection system and the vision based honey bee monitoring systems. The second part describes the techniques used in our experiment and the dataset we used. In the end, we discuss the experiment’s results and we provide suggestions for the further research.

## 2. Related Work

Several studies focus on various honey bee monitoring methods, for example the sound analysis for the swarm detection in [5], bee activity monitoring in [6], or the RFID based individual bee tracking in [7]. A brief overview of other and above mentioned honeybee monitoring techniques can be also found in [8].

Of the image processing based methods, the paper [9] focuses on bee autogrooming phenomena. This study develops a new methodology of modeling the autogrooming process. The bees are covered by baking flour and their grooming is observed by a camera on a controlled background. The speed of the grooming process is automatically analysed.

In [10] the authors try to classify the honeybee brood cells into three categories: occluded, visible and closed, and visible and uncapped for the bee colony self-hygiene monitoring. Authors develop a simple system for the honeycomb’s optical inspection, where they compare the performance of several classifiers. The evaluated classifying algorithms are: support vector machine (SVM), decision tree and boosted classifiers. The best results are obtained with the SVM.

The paper [11] presents a CNN based approach to bee classification based on whether or not the bees are carrying pollen. Hardware setup for image acquisition is described, methods for bee segmentation are discussed and VGG16, VGG19 and ResNet50 CNN performance is compared to classical classifiers such as KNN, Naive Bayes and SVM. Rodriguez et al. conclude that shallower models such as VGG have better performance compared to deeper models such as ResNet. Generally performance of CNN based classifiers was superior to conventional classifiers. What is also important is the discovery that the use of a human predefined colour model, needed for classical classifiers, influenced only the training time but not the final performance of the CNNs when compared to straight RGB input. The authors also created a publicly available dataset [12] with high-resolution images of the pollen bearing and non-pollen bearing bees with 710 image samples in total.

Similarly to the paper above, the dissertation [13] aims for the recognition and tracking of pollen wearing bees. The author uses a Faster-RCNN object detector for the pollen detection on a custom dataset and compares its results with a statistic based methods, which are clearly outperformed by Faster-RCNN. This work contains more valuable results in the bee tracking using Kalman Filtering.

The paper [14] uses a non-invasive hybrid approach to track bees in their natural environment. The proposed algorithm is quite complex, but in a simplified way the authors use a fusion of a background subtraction based detection and YOLOv2 object detector. The algorithm is able to track a single bee in a captured scene and plot its trajectory in a 2D plane, which is an interesting contribution to the state-of-the-art. This work might be useful in the studying of bee behaviour, or its dances in future.

The authors of the article [15] focus on the monitoring of the bees entering and leaving the hive, which could be used for the bee colony health diagnostics as well. For the bee detection, they use a pipeline of a motion detector for an object segmentation and a fusion of the Random Forrest method together with a CNN based image classifier. The authors use previously developed BeePi in-field monitoring system to capture the input data. Two custom datasets created and used in this experiment are made publicly available.

The GitHub repository available at [16] presents a YOLO network trained to recognize between honey bees, wasps and hornets. The results of this experiment are not discussed, or published, but a dataset should be available on request. This work shows the possibility of monitoring bees entering the hive with a common camera.

The investigated V.-mite detection techniques are described in the following articles: The study presented in [17] is focused on detection of V.-mite infestation of honeybee colonies. This study is based on beehive air measurements using an array of partially selective gas sensors in field condition. Collected data was used for training simple LDA and k-NN classifiers with the best misclassification error for k-NN were 17%. The results indicated a possibility to detect honey bee diseases with this approach, but the method must be improved before deployment. In particular, the big and expensive data collection hardware alongside the classifier need to be improved.

The paper [18] shows that the V.-mite-infected brood cells are slightly warmer than the uninfested ones. The experiment was based on the thermal video sensing of brood frames with the artificially infected and uninfected cells in the controlled thermal conditions. The authors observed that infected cells are slightly warmer (between 0.03 and 0.19 ${}^{\circ }$C), which shows the possibility of the thermal measurements in order to detect mite-infected broods.

The article presented in [19] shows a method of monitoring the V.-mite’s motion on the bee brood samples. The authors use classical computer vision methods as background subtraction and geometric pattern analysis with double thresholding to detect the mites on the video frames followed by their tracking on the bee brood with the accuracy rate of 91%. This method is not suitable for in-field-analysis, because the brood must be removed from the cell before the analysis.

The study [20] describes the experimental setup for a non-destructive V.-mite detection and brings suggestions for further development. It also identifies the challenges for mite detection, for example as motion blur, mite colour, or reflections. This paper only briefly describes possible segmentation and detection techniques.

The following article [21] from the same authors describes bee detection and their classification, using classical computer vision methods. In the first part, the authors present good results in segmentation of individual bees using a combination of the Gaussian Mixture Models and the colour thresholding based methods. In the second part, they provide the results of a two class classification (healthy bee/bee with mite) using the Naive Bayes, SVM and Random forest classifiers. The accuracy of these methods varies according to the frame preprocessing techniques and in the best cases moves around 80%.

The last article [22] of the same authors extends the article [21] with the CNN based classifiers (ResNet and AlexNet) and the DeepLabV3 semantic segmentation. The processing pipeline and the classification classes remain the same, as they were in the previous article. Classification of images with a single bee shows good results with the accuracy for the “infected” class around 86%. The experiments with the bees classification from the whole image using the sliding window bring weaker results with the false-negative rate around 67%. The authors also made their dataset publicly available [23].

The study presented in [24] builds on the [11,20,21]. The authors present an entire V.-mite detection pipeline. Firstly, they describe a video monitoring unit with the multispectral illumination, which could be connected directly to the beehive through several narrow pathways. The captured frames are recorded and processed offline, as the bees are segmented with classical computer vision methods and the mites are detected with a custom CNN model. The authors achieved good results in the mite infestation estimation and they proved the potential of the CV based on-site testing.

Practical applications stemming from efficient bee monitoring systems and V.-mite detection systems have also been studied. The authors of [25] investigate the possibility of using a camera based bee monitoring system on the hive’s entrance to detect V.-mites and then destroy them with a focused laser beam. The authors outlined hardware and software requirements for such a system and found it feasible even if such systems have not yet been developed to a deployable state.

## 3. Materials and Methods

In this chapter, we firstly describe insights, our dataset and its statistics. Then follows a brief description of the used detector’s architectures along with the hyperparameters and the evaluation metrics.

The goal of our experiment was to ascertain whether the state-of-the-art object detectors, such as YOLOv5 and SSD, can perform the V. mite and bee detection, alternatively to detect and distinguish between the healthy and the infected bees. Current approaches as [21,24], or [22] use a computationally expensive methods for a bee segmentation (for example Gaussian Mixture Models in [21]), or a bee detection (SIFT and SURF based methods in [24]). All those papers also separate segmentation and classification, which results in a slow processing of the input image.

The SSD and YOLO object detectors do not separate the object segmentation and classification, which results in a possibility of an online processing even on the embedded platforms as NVIDIA Jetson. We believe, that this advantage offsets an expected lower detection accuracy in comparison with a fine tuned segmentation algorithms.

### 3.1. Dataset Description

During the initial phase of our research, we found several publicly available honey-bee datasets. The dataset from [11] is designed for the pollen-wearing bee recognition only and despite its quality and high resolution, it was unsuitable for our task. The dataset presented in [22] contains short videos with mite-infected and unaffected bees in high resolution and it was partially used in our experiment. The dataset [26] seemed promising and brought a lot of additional information, but the images were in a bad quality and low resolution.

**Figure 2:**
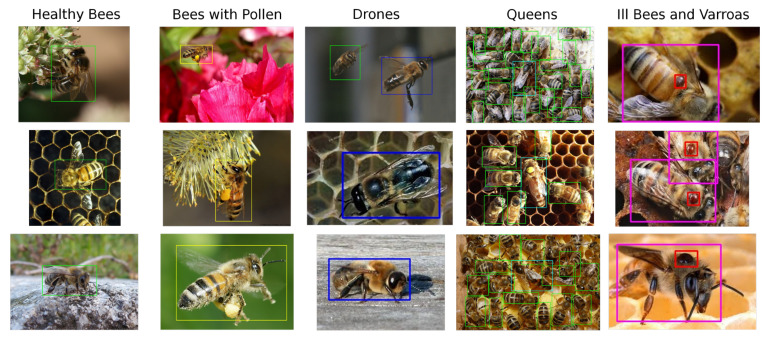
Brief overview of dataset created for the purpose of this work. Healthy bees (green), bees with pollen (yellow), drones (blue), queens (cyan), infected bees (purple), V.-mite (red).

For the above reasons, our custom dataset was compiled from publicly available images and partially from the dataset [23]. It contains a total of 803 unique samples, where 500 samples capture bees in the general environment and the rest, taken from the dataset [23], shows bees on an artificial background. Dataset samples can be seen at Figure 2.

**Figure 3:**
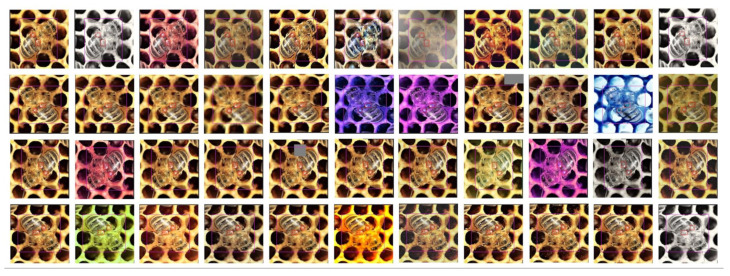
Example of a single image (left top) augmented into the ten more training samples. In the top left column, there is the original image rotated by 0, 90, 180, and 270 degrees. In the rest of the row, there are the images augmented by the ImgAug framework.

In our dataset and with future experimentation in mind, we define six classes shown in Figure 3, the healthy bee (1), the bee with pollen (2) (pollen may be miss-classified with a V.-mite), the drone (3), the queen (4), V.-mite-infected bee (5), and the V.-mite (6). However, only for this paper, we reduced the data annotation into three different subsets. The first one is the bees (classes 1, 2, 3, 4, 5) and V.-mite (class 6), the second one is the healthy bees (classes 1, 2, 3, 4) and infected bees (class 5), finally in the last subset are the data annotated only with V.-mite (only class 6).

We created these three derivations of the original dataset annotation to test, which one will give us the best result in detecting varroosis infection in the beehive. Our dataset was manually annotated with the LabelImg tool [27], and its statistics are presented in Table 1, Table 2, Table 3 and Table 4. All annotations were consulted with a professional beekeeper.

Even though we used a part of the images from the dataset [23], the number of gathered images to train the neural network was not sufficient. Therefore we have decided to apply an augmentation to the entire training subset. Generally speaking, augmentation helps increase the robustness of the training process, helps to avoid overfitting, and overall improves the neural network’s performance at the end of the training phase [28].

To apply augmentation to our data, we have used the Python library called ImgAug [29]. It provides a wide range of various methods to augment the image by blurring, adding different noise signals, various effects (like motion, fog, rain, color space alteration, etc.), random erasing (Random Erasing Data Augmentation) bounding box modifications, or even the color space modifications and shifting or image cutouts [28,30].

The main idea of the augmentation method is to create slightly modified derivatives of the original training data that, even after modification, still represents the original problem. In the case of object detection or, generally speaking, computer vision, by applying rotation, geometrical distortion, additional noise, or the colour shift, we do not modify the information that the image contains. Later, during the training process, the neural network is forced to learn how to solve the given task for perfect-looking training data as well as, for distorted, noise-added, or colour shifted images. It makes models to better generalize problems, and the entire learning process is way more robust against overfitting.

In the case of V.-mite detection, we can illustrate the problem of the close similarity between the V.-mite and the bee’s eye. Both objects are very close to each other in their geometrical shapes and dimensions. The differences here are the colour and the close surroundings. The mite is strictly brown whereas the bee’s eyes are black. If we let the neural network to train on the unaugmented images, it could learn to identify mites only by the presence of the brown colour. If the images are augmented, the neural network has to understand the entire structure of the mite body.

In our case, we rotated each image by 90, 180, and 270 degrees, and for every rotation, we have created ten derivatives of images by applying a single, randomly selected augmentation style from the available set. Using this method, we had augmented every single image from the original training set and we created additional 43 samples. It is important to note that the validation and the test set stay without any modifications, so they represent the bees and V.-mites real-life image data.

In total, we created the dataset containing 803 images. The training set contains 561 images, later augmented for the total of 24,684 images, 127 images comprise the validation set, and the test set contains further 115 samples. All images in the original 803-images set are independent of each other. For those 803 images, we created three annotations as mentioned before, and the number of annotated instances in each class for training, validation and test set is in Table 1, Table 2, Table 3 and Table 4.

We are aware that for image classification tasks it is crucial to have a balanced dataset for all classes. In case a balanced dataset is not available, the architecture will not be able to train classifying the underrepresented classes. In order to identify this potential problem in our slightly unbalanced dataset, we use the mAP[0.5] score as one of our metrics. This score is affected by all classes with the same weight and it is described in more detail below.

### 3.2. Network Description

In this section a brief description of the YOLOv5, SSD object detectors and the Deep SVDD anomaly detector is provided as used in our experiment.

#### 3.2.1. YOLOv5

The original YOLO [31] and all its derivatives (YOLO9000, YOLOv3, YOLOv4) are examples of end-to-end object detection models. It means the inference of the network with the image is the only operation performed during the object detection. There is nothing like region proposals, combination of detected bounding boxes etc. [32]. The YOLO architecture has only the image on the input and the vector of detections and probabilities of these detections on the output.

In this paper, we have used the open-source implementation of the YOLO object detector, called YOLOv5 from Ultralytics, available from [33]. The YOLOv5 is not a self-standing version, that would bring significant improvements in the YOLO-like neural networks architectures, more it is one example of implementing the YOLOv3 principles in the PyTorch framework [34]. Also, as of the day of writing this paper, there is no official peer-reviewed article about YOLOv5.

The YOLOv5 consists of three main parts. The backend is a standard convolutional backend as we know it from other neural networks (VGG, ResNet, DarkNet, etc). The backend extracts the feature maps from the input image and performs the geometrical pattern detections. As we go deeper through the backend, the extracted feature maps’ resolution is decreasing, and the neural network detects larger and more complex geometrical shapes.

The second part is the “neck” stage. It takes the feature maps from several backend levels (different backend levels detect objects of different sizes and geometrical complexities) and combines them into three output scales.

The last part is the prediction park that uses 1 × 1 convolution layers to map different scales of concatenated feature maps from the “neck” stage into three output tensors.

There exist two variants of the YOLOv5 architecture, the S and the X model. The difference between the YOLOv5 S and X versions is in the dimensions of the neural networks. The X version multiplies the number of kernels per convolutional layer by 2.5 w.r.t. the S variant and the number of convolutional layers are multiplied by 4. Comparing the S and X models by numbers, the X version has 476 layers, 87.7 million parameters, and the S version has 224 layers and 7.2 million parameters. The detailed inner structure overview can be found on the project’s Github webpage [33].

#### 3.2.2. SSD

The Single Shot Multibox Detector (SSD) is a fast object detector, originally presented in [35]. The architecture consists of a feature extracting base-net (originally VGG16) and classifying layers. The base-net findes feature map using convolutional layers, with the number of features rising as we go deeper into the base-net. The classifying layers are connected to the base-net on several levels for different level features. For this experiment, we used the open source implementation available from [36].

The SSD detector processes images or batch of images through net and compute output. The output is represented by the predicted object’s location bounding box and confidence. For this output the architecture generates tens of thousands of priors, which are base bounding boxes similar to anchor boxes in Faster RCNN [32] from which we compute regression loss.

This architecture creates the base net and adds several predictors for finding different scales of objects. As we go deeper into the base-net, we assume finding a bigger object as the feature map corresponds to a bigger receptive field. The first predictor finds boxes after 14 convolution layers, therefore fails to find very small objects (feature map 38 × 38 px).

In our experiment, we use two base-nets. The first one is the original VGG16 and the second in MobileNet v2. The main difference is in the network’s complexity (VGG16 has approx 138 mil. parameters and MobileNet v2 has approx. 2.3 mil.).

#### 3.2.3. Deep SVDD

The Deep Support Vector Data Description (Deep SVDD) is a convolutional neural network based anomaly detector (AD) presented in the [37]. It is designed for the detection of anomalous samples (one class classification) and it already brought us good results on homogenous datasets.

This technique searches for a neural network transform, which maps the majority of the input data to a hypersphere with a center c and a radius R with a minimal volume. The samples, which fall into this area are considered as normal and the samples out of the sphere as anomalous. The center of the hypersphere is computed from the mean features of the all input samples and the decision boundary (parameter R) is set by a threshold. The input data might be processed by an autoencoder. For our experiment, we used the implementation available from [38].

We used this model in our experiment even though it is not an object detector, because we wanted to prove whether this architecture yields good results in the infected bee detection. As we mentioned in the introduction, the V.-mite infested bees are often deformed and the parasite might not be always visible, or present on the bee’s body. With the AD technique based approach, we could be able to detect those cases, or even bees with other problems.

For this experiment, we used 200 samples from the dataset [23], because this method requires similar looking samples, which could not be sourced from our part of the dataset described above.

### 3.3. Hyperparameters

We trained all networks on the Nvidia GTX 2080 GPU card and all networks were trained on a 640 by 640 px images. For the YOLOv5 the batch size was four images. We used the ADAM optimizer. For the SSD we use batch size with ten images and SGD optimizer with momentum of 0.9. For the YOLOv5 and SSD networks, we used the implementation’s default pretrained weights to boost the learning process and save computational resources. In all cases, we left all network weights unlocked for training.

We trained each YOLO model for 100 epochs and the performance of the models saturated after 30 epochs. The SSD models were trained again for 100 epochs and they saturated after 40 epoch. The probability threshold was set to 0.4 for YOLO and 0.3 for SSD.

We let the defaults anchor boxes for the YOLOv5 model: [10,13, 16,30, 33,23] (P3/8), [30,61, 62,45, 59,119] (P4/16) and [116,90, 156,198, 373,326] (P5/32), as defined in [33]. For the SSD model, the anchor boxes required more tuning and we set the for both base nets as described in Table 5:

The size of the SSD anchor boxes was set according to the size of the V-mites in our dataset, which varied in range between 15–25 px.

### 3.4. Used Metrics

To estimate the performance of the artificial intelligence models, we need to define metrics that will give us an idea of how well the model could solve a given problem after the training process.

One of the most common ways to express object detection capability to perform well is the mean average precision (mAP) metrics first introduced by the [39] as the mAP[0.5]. This metric denotes the relative number of object detection precision using the minimal intersection over union (IoU) with a value equal to or bigger than 0.5.

Another variant of the mAP metrics is the mAP[0.5:0.95], first used by [40]. In addition to mAP[0.5], mAP[0.5:0.95] calculates the average for all mAP values with the IoU level 0.5 up to 0.95 with 0.05 step.

**Figure 4:**
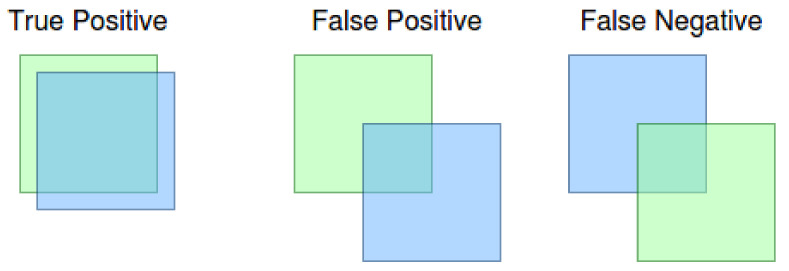
True positive, false positive and false negative results.

The meaning of true positive, as well as false positive and false negative in context of the IoU metrics can be seen in Figure 4. As can be seen in the figure, the result is a true positive if the ground truth (blue bounding box) and the detection (green bounding box) has a level of IoU at least 0.5. We obtain the false positive result if the neural network declares the detection, and there is no ground truth bounding box with at least 0.5 IoU. The false negative result means that the ground truth bounding box has no detection to match with at least 0.5 IoU. Note: the x value defines 0.5 IoU from mAP[x] metric.

Then the precision and recall are given by:

(1) $$precision=\frac{TruePositive}{TruePositive+FalsePositive}recall=\frac{TruePositive}{TruePositive+FalseNegative}$$

Given something, we define the usual statistical metrics called the *F*1 score as:

(2) $$F1=2*\frac{precision*recall}{precision+recall}$$

## 4. Results

### 4.1. YOLOv5

**Figure 5:**
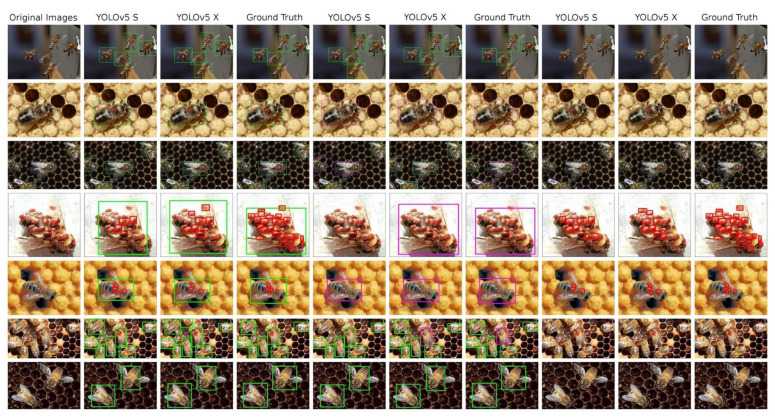
An overview of the detections performed on test images by the YOLO models, complemented by ground truth annotated images. 1st column: original image; 2nd–4th column—model trained in bees and V.-mites detection; 5th–7th column: models trained in healthy and ill bees detection; 8th–10th column: models trained in V.-mite detection only.

The Table 6 presents all performance numbers reached by YOLOv5 models trained on our dataset. All six models are always the “S” and “X” variant of the YOLOv5, trained on three differently annotated modifications of the original data—the bees and V.-mite detection, the healthy and ill bees detection and the V.-mite only detection. Detection results can be seen at Figure 5

The primary purpose of our work is to track the health of the beehive population. From this perspective, the best results are shown by the X model trained on the Healthy and Ill bees dataset. It has the highest F1 score that expresses the relation between the precision and recall performance when detecting bees influenced by the V.-mite, but it also gives the best results from the mAP point of view where it outperforms other models trained on the same images but with different annotation.

Originally, we expected that the recognition of the tiny difference between the healthy bee and the one infected by the V.-mite would be the most challenging problem for the neural network. In the end however, the opposite was proven to be true.

### 4.2. SSD

**Figure 6:**
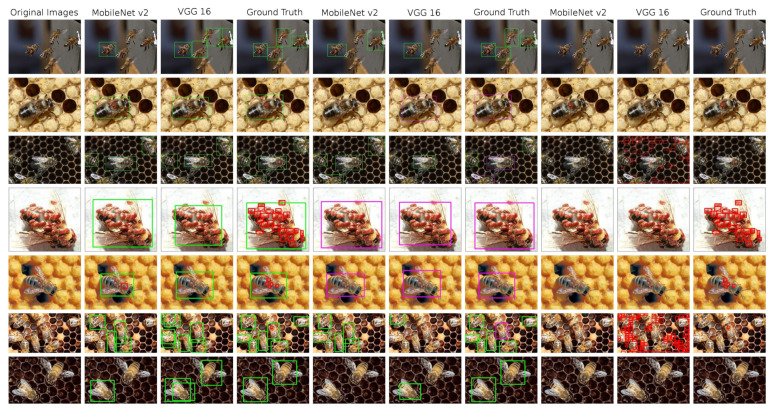
An overview of the detections performed on test images by the SSD models, complemented by ground truth annotated images. 1st column: original image; 2nd–4th column—model trained in bees and V.-mites detection; 5th–7th column: models trained in healthy and ill bees detection; 8th–10th column: models trained in V.-mite detection only.

SSD object detector results are presented in the Table 7. Just as in the previous experiment, we tested three datasets and two base nets. The shortcut mb2 stands for the MobileNetV2 and the shortcut vgg stands for the VGG16 architecture. Detection results can be seen at Figure 6

SSD performs worse than YOLOv5 in almost all metrics (with the exception of the F1 score on the V.-mites dataset). However the results show a slight potential of this detector for our task, when better results might be achieved by fine tuning and a further experimentation with the detector’s anchor boxes. As the MobileNetV2 is more recent and higher performance architecture, it clearly outperforms the VGG16 base net in all categories, especially on the V.-mites dataset.

### 4.3. Deep SVDD

Beside the YOLOv5 and SSD object detectors, we tried to use the Deep SVDD anomaly detector [37] on the part of the dataset [23]. Using this approach, we expected a better recognition of the bees deformated by the V.-mite, but this method didn’t bring any satisfactory results. Possible explanations of the failure of this method could be, that the image features are too close to each other, or that the anomalous bees create a cluster inside the correct features.

## 5. Discussion

To compare the performance of both object detectors, we decided to use mAP[0.5] and F1 scores. The mAP[0.5] is a commonly used standard of object detector’s performance measurement. We selected the mAP[0.5] over the the mAP[0.5:0.95] as the latter metric is highly influenced by the size of detected objects and for smaller objects (V.-mite) it gives a significantly worse score than for large objects (bees). As the second metric, we chose F1 score as an expression of the relation between the precision and the recall, as we need both of these parameters to have high values to avoid false positives and false negative detections.

The outcomes shown above prove that the object detectors can be used in the visual honey bee health state inspection and that our results are comparable with the other related research. Based on our results, the YOLOv5 object detector clearly outperforms the SSD in all but one cases. An overview of our results is shown in Table 8.

The best results were obtained with the YOLOv5 neural networks trained on the Healthy and Ill bees dataset. In the S variant, YOLOv5 reaches 0.902 mAP[0.5] and 0.838 F1 score. For the X variant it reaches 0.908 mAP[0.5] and 0.874 F1 score. Obtained F1 score on the Bees and V.-mites dataset is lower than the comparable scores 0.95 and 0.99 achieved in [22,24]. We explain this difference with a higher difficulty of the object detection task in comparison with the object classification and with the use of a more complex dataset than in the papers above. Nevertheless, the resulting F1 score on the Healthy and Ill bees dataset is comparable to the mentioned papers. In our opinion, distinguishing between the two classes can prove even more robust than the simple V.-mite detection, because the infection is often connected with the bee body deformities and the V.-mite itself might not always be present on the bee’s body.

The SSD detector didn’t fulfil our expectations and performed worse than the YOLOv5. We explain this issue with the older and more complicated architecture of this detector, which makes it more difficult to tune. The MobileNetV2 consistently outperforms the VGG16 base net and gives the best F1 score (0.714) on the V.-mite dataset. From the speed test, the MobileNetV2 showed faster interference than the VGG16, probably because of the network’s complexity, e.g., number of trainable parameters. Based on these findings, we will focus more on the YOLOv5 architecture in our future experiments.

The proposed methodology for finding V.-mites has its limitations, especially in the field of computational complexity, which requires powerful hardware. All ours experiments were performed on a computer workstation with dedicated graphics card. The recent developments in the field of embedded platforms (such as NVIDIA Jetson) also allows their use for the deep learning algorithms [41], and promise significant increase in embedded hardware capabilities.

The accuracy of our approach is slightly inferior to the approaches based on the conventional CV methods or their fusion. We explain this fact with two reasons: Firstly, we used a more difficult dataset with the bees in both controlled and general environments to demonstrate the robustness of our approach. Secondly, the classical methods might be more easily tuned to the given problem, but they show a worse generalization. As was described in the beginning of Section 3, the main benefit of our approach is a possibility of the online processing. This could be very useful for the bee colony health state inspection device, where the beekeeper would see the results instantly. This device also wouldn’t have to be connected to a remote server to perform the inspection and could be further extended for other tasks, such as pollen, wasp, or drone detection. We also see a potential for further precision improvement by retraining of the models on a greater dataset obtained on the inspection site. We believe, that the disadvantage of a lower accuracy is outweighed by the advantages described above and that this issue could be further improved by retraining on a larger dataset.

Table 9 shows the average inference time for the Nvidia 1080 Ti and 2080 Ti GPUs for all neural network models used in this paper. The 1080 Ti GPU has a slightly higher core frequency, 1480/1582 MHz (1080 Ti) compared to 1350/1545 MHz (2080 Ti) [common/max freq.], but 2080 Ti has a higher number of CUDA cores, 3584 cores (1080 Ti) compared to 4352 cores (2080 Ti). That setup makes 1080 Ti GPU faster for inference over models that provide fewer opportunities to parallelize computation, like VGG16, when 2080 Ti processes more parallelized models, like YOLOv5 X, much faster.

In the future, we plan to deploy models on the Jetson Nano embedded computer. It provides 128 CUDA cores with a core frequency of 1479 MHz. It makes Jetson Nano approximately 30 times slower compared to the 1080 Ti and 2080 Ti. In this case, the Jetson Nano should inference the YOLOv5 X version at approximately 1 FPS, which is sufficient for our purpose.

## 6. Conclusions

Our paper presents another approach with an online measurement potential to identify V.-mite infection in the beehive by using object detectors. To the best of our knowledge, there is no other paper that deals with the Varroosis detection problem this way, as most of the papers are studying only image classification. Moreover, we split our work into three directions by modifying the datasets to train three slightly different problems. The first variant was to train neural networks to detect both bees and V.-mite. The second approach was to detect healthy and V.-mite infested bees, and the third approach was to detect V.-mite only.

We tested three architectures, the YOLOv5 neural network, the SSD neural network (both object detectors) and the SVDD, a neural network-based anomaly detector. The YOLOv5 and SSD object detectors proved to be suitable for this task, however, the SVDD anomaly detector was not able to model the problem. We plan to use both YOLOv5 and SSD detectors in our future experiments.

Unlike the papers mentioned above, the YOLOv5 and SSD inference times should allow us to process the inspection results online. We plan to use this ability in our future work, where we would like to develop a monitoring system similar to the [24] with the emphasis on low cost and it being a more portable solution. We also plan to extend our dataset in order to monitor other bee infections and bee colony activities.

## Figures and Tables

**Table 1:** The table shows the number of annotated objects of given classes.

**All Classes** **Dataset**	**No. of Annotated Objects per Class**						**Images**
	**Bee Worker** **(No Pollen)**	**Bee Worker** **(Pollen)**	**Bee** **Drone**	**Bee** **Queen**	**Bee with** **V.mite(s)**	**V.-Mite**	
	**1158**	**143**	**19**	**52**	**298**	**424**	**803**

**Table 2:** The table shows the number of annotated objects of given classes of the **Bees and varroosis** dataset.

Bees and V.-mites Dataset	Classes	Bees	V.-Mite	Images
	Train Set	1148	250	561
	Train Aug Set	50,512	11,000	24,684
	Val Set	274	92	127
	Test Set	248	59	115

**Table 3:** The table shows the number of annotated objects of given classes of the **Healthy and Infected bees** dataset.

Healthy and Ill Bees Dataset	Classes	Healthy Bees	Infected Bees	Images
	Train Set	956	192	561
	Train Aug Set	42,064	8448	24,684
	Val Set	220	54	127
	Test Set	196	52	115

**Table 4:** The table shows the number of annotated objects of given classes of the **Varroosis only** dataset.

V.-Mites Dataset-	Classes Train Set	V.-Mite 250	Images 561
	Train Aug Set	11,000	24,684
	Val Set	92	127
	Test Set	59	115

**Table 5:** Setting of the SSD anchor boxes.

Base Net	Feature Map Size	Shringkage	Anchor Box	Aspect Ratio
VGG 16	80	8	(15, 30)	[2, 3]
	40	16	(30, 60)	[2, 3]
	20	32	(60, 105)	[2, 3]
	10	64	(105, 150)	[2, 3]
	8	80	(150, 200)	[2, 3]
	6	107	(250, 340)	[2, 3]
MobileNet v2	40	16	(15, 30)	[2, 3]
	20	32	(30, 60)	[2, 3]
	10	64	(60, 105)	[2, 3]
	5	128	(105, 150)	[2, 3]
	3	214	(150, 200)	[2, 3]
	2	320	(200, 340)	[2, 3]

**Table 6:** Test score achieved by the YOLOv5 models on all datasets variants. The best results in V.-mite detection achieved by the YOLOv5 X model for the Healthy and Ill bees dataset.

Bees and Varroa Mites Annotated Dataset										
Metrics	mAP[0.5]		mAP[0.5:0.95]		F1		Precision		Recall	
Yolov5 model	S	X	S	X	S	X	S	X	S	X
Bees	0.953	0.946	0.610	0.647	0.827	0.859	0.711	0.762	0.989	0.985
V.-Mites	0.649	0.726	0.260	0.276	0.638	0.650	0.657	0.609	0.620	0.696
Average	0.801	**0.845**	0.435	**0.462**	0.733	**0.754**	0.684	**0.686**	0.805	**0.841**
**Healthy and Ill Bees Annotated Dataset**										
Metrics	mAP[0.5]		mAP[0.5:0.95]		F1		Precision		Recall	
Yolov5 model	S	X	S	X	S	X	S	X	S	X
Healthy Bees	0.932	0.938	0.589	0.676	0.826	0.865	0.722	0.824	0.964	0.910
Ill Bees	0.902	0.908	0.726	0.746	0.838	0.874	0.825	0.902	0.852	0.848
Average	0.917	**0.923**	0.658	**0.711**	0.832	**0.870**	0.774	**0.863**	**0.908**	0.879
**Varroa Mites Annotated Dataset**										
Metrics	mAP[0.5]		mAP[0.5:0.95]		F1		Precision		Recall	
Yolov5 model	S	X	S	X	S	X	S	X	S	X
V.-Mites	**0.777**	0.752	**0.328**	0.327	0.656	**0.666**	0.585	**0.626**	0.746	**0.712**

**Table 7:** Test score achieved by the SSD models on all datasets variants. The best results in V.-mite detection achieved by the SSD X model for the Healthy and Ill bees dataset.

Bees and Varroa Mites Annotated Dataset										
Metrics	mAP[0.5]		mAP[0.5:0.95]		F1		Precision		Recall	
Base Net	VGG	MB2	VGG	MB2	VGG	MB2	VGG	MB2	VGG	MB2
Bees	0.354	0.547	0.234	0.281	0.529	0.556	0.920	0.512	0.371	0.609
V.-Mites	-	0.529	-	0.252	-	0.681	-	0.914	-	0.542
Average	-	**0.533**	-	**0.266**	-	**0.619**	-	**0.713**	-	**0.576**
**Healthy and Ill Bees Annotated Dataset**										
Metrics	mAP[0.5]		mAP[0.5:0.95]		F1		Precision		Recall	
Base Net	VGG	MB2	VGG	MB2	VGG	MB2	VGG	MB2	VGG	MB2
Healthy Bees	-	0.395	-	0.203	-	0.520	-	0.541	-	0.5
Ill Bees	-	0.182	-	0.114	-	0.318	-	0.909	-	0.192
Average	-	**0.288**	-	**0.159**	-	**0.419**	-	**0.725**	-	**0.346**
**Varroa Mites Annotated Dataset**										
Metrics	mAP[0.5]		mAP[0.5:0.95]		F1		Precision		Recall	
Base Net	VGG	MB2	VGG	MB2	VGG	MB2	VGG	MB2	VGG	MB2
V.-Mites	0.355	**0.519**	0.184	**0.239**	0.242	**0.714**	**0.980**	0.897	0.322	**0.593**

**Table 8:** Table shows the mAP[0.5] score at V.-mites detection (V.-mites or an infected bee) for all neural network models that we used in this work.

Detecting V.-Mite Presence								
	**mAP[0.5]**				**F1 Score**			
Training Dataset	YOLOv5 S	YOLOv5 X	SSD-VGG	SSD-MobNv2	YOLOv5 S	YOLOv5 X	SSD-VGG	SSD-MobNv2
Bees and V.Mites (V.-mite score only)	0.649	0.726	-	0.529	0.638	0.650	-	0.681
Health and Ill Bees (ill bees score only)	0.902	0.908	-	0.182	0.838	0.874	-	0.318
V.-Mites Only	0.777	0.752	0.355	0.519	0.656	0.666	0.242	0.714

**Table 9:** Inference time (in seconds) of the used object detectors on the NVIDIA 1080 Ti and NVIDIA 2080 Ti GPUs.

Neural Netoworks Inference Duration [ms]				
GPUs	YOLOv5		SSD	
	S	X	VGG	MobNetv2
1080 Ti	0.045	0.011	0.665	0.109
2080 Ti	0.031	0.012	0.932	0.106

## Data Availability

Not applicable.
